# Differential Proteomics Analysis of Colonic Tissues in Patients of Slow Transit Constipation

**DOI:** 10.1155/2016/4814702

**Published:** 2016-04-30

**Authors:** Songlin Wan, Weicheng Liu, Cuiping Tian, Xianghai Ren, Zhao Ding, Qun Qian, Congqing Jiang, Yunhua Wu

**Affiliations:** Zhongnan Hospital of Wuhan University, Department of Colorectal & Anal Surgery, Clinical Center of Intestinal and Colorectal Diseases of Hubei Province, Key Laboratory of Intestinal & Colorectal Diseases of Hubei Province, Wuhan University, No. 169, Donghu Road, Wuchang District, Wuhan, Hubei 430071, China

## Abstract

*Objective*. To investigate and screen the different expression of proteins in STC and normal group with a comparative proteomic approach.* Methods*. Two-dimensional electrophoresis was applied to separate the proteins in specimens from both 5 STC patients and 5 normal controls. The proteins with statistically significant differential expression between two groups were identified by computer aided image analysis and matrix assisted laser desorption ionization tandem time of flight mass spectrometry (MALDI-TOF-MS).* Results*. A total of 239 protein spots were identified in the average gel of the normal control and 215 in patients with STC. A total of 197 protein spots were matched and the mean matching rate was 82%. There were 14 protein spots which were expressed with statistically significant differences from others. Of those 14 protein spots, the expression of 12 spots increased markedly, while that of 2 spots decreased significantly.* Conclusion*. The proteomics expression in colonic specimens of STC patients is statistically significantly different from that of normal control, which may be associated with the pathogenesis of STC.

## 1. Introduction

Constipation is a very common functional gastrointestinal disorder that affects patients' quality of life [1, 2]. The prevalence of constipation in the worldwide general population is very variable, ranging from 2% to 35% in adults and ranging from 0.7% to 29.6% in children [3]. Slow transit constipation (STC) is common type of chronic constipation, which affects 13–37% of patients who have chronic, treatment-resistant constipation [4, 5]. The epidemiological data show that there is a higher incidence of STC in young females than in males [6, 7].

STC is characterized by slow proximal colonic transit and prolongation of transit time through the colon, which can be demonstrated with radiopaque marker transit tests [8, 9]. Despite its high prevalence, its etiology and precise mechanism(s) remain unknown [4, 10], and STC have become the main subject of many colorectal and anal surgeries. Although several morphological changes have been reported, the exact mechanism(s) of STC pathogenesis remains poorly understood and there are no effective clinical treatments. Even if some patients' clinical symptoms may improve after conservative management, the small subset of STC patients who do not respond to conservative management are considered candidates for surgery [11].

At present, quantitative proteomic analysis has become an important approach to screen the key protein of the pathogenesis of diseases. “Proteome” first referred to the total protein complement encoded by a given genome. Now it comprises any isoforms, posttranslational modifications, interactions, and actually everything “postgenomic” [12, 13]. The research about proteomics involves large scale detection, identification, and characterization of proteins, which is highly promising for biomarker detection over many diseases [14, 15]. The most common method applied is a combination of two-dimensional electrophoresis (2DE) and mass spectrometry.

In our study, we separate protein in test group and control group through two-dimensional electrophoresis (2DE), ImageMaster 2D Elite computer aided image analysis, and matrix assisted laser desorption ionization tandem time of flight mass spectrometry (MALDI-TOF-MS), to find out the key protein acting in the pathogenesis of STC.

## 2. Materials and Methods

### 2.1. General Information

Patients were divided into a test group and a control group; each group included 5 cases. All patients had undergone a rigorous selection. First, all candidates should have prior screening through repeated gastrointestinal transit time (GITT) study, using 20 radiopaque markers, and abdominal imaging tests performed on 6 h, days 1, 2, and 3. And the abdominal imaging test performed on 6 h after taking 20 radiopaque markers was used to identify whether there was slow transit in the terminal intestine. All these five STC patients showed slow transit mainly on the left colon through GITT study and all samples were collected from the left hemicolon, and the same regions were available for both STC and control groups. Second, autoimmune diseases such as diabetes mellitus type I, autoimmune enteric leiomyositis, rheumatoid arthritis, systemic sclerosis, and systemic lupus erythematosus can prolong colonic transit time by damage of colonic smooth muscle cells. And all candidates with any of these autoimmune diseases were excluded and all these five STC patients selected were without any autoimmune diseases at all. Other evaluations including colonoscopy, barium enema, defecography, and anorectal manometry were used to rule out colorectal neoplasms, megacolon, outlet obstruction, and pelvic floor dysfunction. The conventional medical therapy had failed in all STC patients. Conservative treatment did not improve their bowel habits, and defecation frequently had to be achieved by the application of stimulus laxatives and enemas. All STC patients (*n* = 5, mean age 50 years, range 43–81 years; 5 women and 0 men) underwent subtotal colectomy with antiperistaltic cecoproctostomy or total colectomy with ileorectal anastomosis. Patients in the control group (*n* = 5, mean age 52 years, range 41–79 years; 5 women and 0 men) underwent partial colectomy for incomplete intestinal obstruction caused by colorectal neoplasms and the control tissue was taken from the proximal end. All control patients were reported with normal bowel habits. All specimens were obtained immediately after resection and snap-frozen in liquid nitrogen until being used.

The high-abundance proteins were removed with the steps recommended by Calbiochem Company and the improved Bradford method was used for sample protein quantitation [16].

### 2.2. Two-Dimensional Electrophoresis (2D)

#### 2.2.1. Extraction and Determination of the Concentration of Protein

A 2.0 mL sterile EP tube was prepared. About 200 mg small piece of tissue was rapidly clipped from the specimen and weighed on electronic scale, and then the tissue was placed in the precooling Petri dish with normal saline (6 mL, 0.9% NaCl). The tissue was shredded in the dish and then transferred to 1.5 mL EP tube. 800 *μ*L cold protein lysate (containing a protease inhibitor) was added into the EP tube which was to be quickly transferred to an ultrasonic homogenizer. The EP tube was coated with ice. The ultrasonic homogenizer was triggered 5 times, 3 seconds each time. The tube was statically placed for 30 minutes; the supernatant was collected by centrifugation (4000 r/min 4°C, 60 min) and then stored in the −80°C refrigerator.

#### 2.2.2. Protein Purification

The protein sample was transferred to a labeled EP tube (2 mL). After adding protein cracking fluid with 3 times volume of samples, the mixture was shock-cracked and subsequently placed on ice for 15 min. Then, the mixture was centrifuged for 8 min with a high speed of 12000 r/min at 10°C. The supernatant was discarded and the precipitation was resuspended and was shock-cracked for 30 s every 10 min for 3 cycles. Finally, the precipitation was resuspended again and used for next steps.

#### 2.2.3. Concentration Determination

Protein concentrations were determined by the instruction of the Bio-Rad RCDC kit manual. The specific steps were as follows: standard concentration and gradient BSA reagent were prepared and the concentrations were, respectively, 0, 0.25, 0.5, 0.75, 1, 1.25, and 1.5 (g/mL). Reagent A working fluid resulted from the mix of 20 *μ*L DC Reagent S and 1 mL DC Reagent A. Eight 1.5 mL EP tubes were, respectively, added with 25 *μ*L standard BCA solution and 5-fold dilution of the sample, and then in each tube 125 *μ*L RC Reagent I and RC Reagent II were added. The mix was collected by centrifugation (15000 r/min, 5 min) after the supernatant was discarded. 127 *μ*L Reagent A was added to the tubes. The tubes were statically placed for 5 minutes after blending. Then, 1 mL DC Reagent B was added to each tube and blended. Absorbance values (750 nm) were measured after statically placed at room temperature for 15 min.

#### 2.2.4. Isoelectrofocusing (IEF)

Rehydration stock solution with IPG buffer was dissolved at room temperature (DTT and Bio-Lyte were added before using). The proper amount of rehydration stock solution with IPG buffer was added to samples till a final volume of 480 *μ*L according to the concentration of the samples. The sample was linearly added along with the extension of the focusing plate. The protective layer on the surface of the gel was stripped with tweezers so as to distinguish positive and negative electrodes, and then the gel was placed downside onto the sample solution in the focusing plate. 2 mL mineral oil was covered by each gel. The mineral oil was slowly added onto the support film drop by drop. IEF was carried out at 50 V (14 h), 250 V linear (30 min), 1000 V fast (60 min), 10000 V linear (1 h), 10000 V fast 80000 V, and 500 V (arbitrary time).

#### 2.2.5. SDS-PAGE Electrophoresis

Two 10% acrylamide gels were prepared. 1 cm was reserved on the upper side and covered with water-saturated n-butanol and then polymerized for 30 min. The n-butanol was then discarded. The gels were washed three times with ultrapure water. Water on the glass plate was sucked with filter paper. The gels were taken to the hydration plates, and 6 mL equilibrium liquid I (0.2 g DTT per 10 mL equilibrium mother liquid before using) and II (0.25 g iodoacetamide per 10 mL equilibrium mother liquid before using) were then added into each plate. The plate was placed on a shaker (15 min). The strip was carefully placed on the surface of the gel. Air bubble between them was removed. The heated sealant was cooled to 37°C and then applied to the surface of gel. The gel was transferred to the electrophoresis tank after the sealant was solidified. The upper electrophoresis tank was filled with electrophoresis solution after checking the positive and negative charges. The electrophoresis was first carried out at Low Voltage (5 mA/gel) within 2 cm downside the sample, and then the electrophoresis was carried out to the bottom of the sample with a current of 30 mA/gel.

#### 2.2.6. Coomassie Blue Staining

Gels were dyed with Coomassie Blue for 16 h after being fixed with 12% TCA for 2 h. Then, the gels were washed with 0.1 mol/L Tris-H_3_PO_4_ (pH 6.5) for 2 min and then with 25% alcohol for 1 min at most. At last, the gels were fixed with protein-dye compounds again.

#### 2.2.7. Gel analysis

After scanned by Image Scanner II transmission scanner and marked with Scan scanning software, the gels were analyzed by image analysis software (ImageMaster 2D Elite5.0). And plots with expression differences more than 10 times (*P* < 0.05) were chosen as targeted proteins.

### 2.3. Mass Spectrum Identification

#### 2.3.1. Enzymatic Hydrolysis

Targeted parts of the gels were removed and washed with ultrapure water and then decolorized with 200 *μ*L mixed liquid (25 mmol/LNH_4_HCO_3_, 50% ACN) for 20 min at 37°C. An alternative procedure was ultrasonic decoloring. Then, 100 *μ*L ACN was added in the mixture and discarded when the color of the gels turns white. Subsequently, the gels were enzymatically hydrolyzed with Trypsin (diluted with 25 mmol/LNH_4_HCO_3_ at a concentration of 12.5 mg/mL) for 45 min at 4°C. Finally, gels were continuously hydrolyzed with 25 mmol/LNH_4_HCO_3_ 10 *μ*L at 37°C for 16 h.

#### 2.3.2. Mass Spectrometry Analysis

Substrate (4 mg HCCA in 1 mL solution of (ACN: 0.1% TFA (70 : 30))) was prepared; the enzymatically hydrolyzed gels were dried and covered with 0.1 *μ*L substrate. Then the mixture was diluted 25 times its original volume and salts were removed using 0.1% TFA.

#### 2.3.3. Database Analysis

Data from mass spectrometry were analyzed and screened with Swiss-Prot Database in Mascot.

### 2.4. Statistical Analysis

Statistical analyses were performed using SPSS 19.0 software for Windows (SPSS, Chicago, IL, USA). All groups of variables were tested for normal distribution using the Kolmogorov-Smirnov test and normally distributed data were expressed as mean ± SD, followed by a two-tailed Student's *t* test to determine *P* values. All results with *P* < 0.05 were considered statistically significant.

All research involving human participants was approved by the Zhongnan Hospital of Wuhan University Ethics Committee, and we obtained written informed consent from all participants before they were enrolled in the study (ethical considerations: ethics number: 2010010; ethical approval starting date: March 8, 2010; ethical approval expiration date: March 8, 2013).

## 3. Results

**Figure 1:**
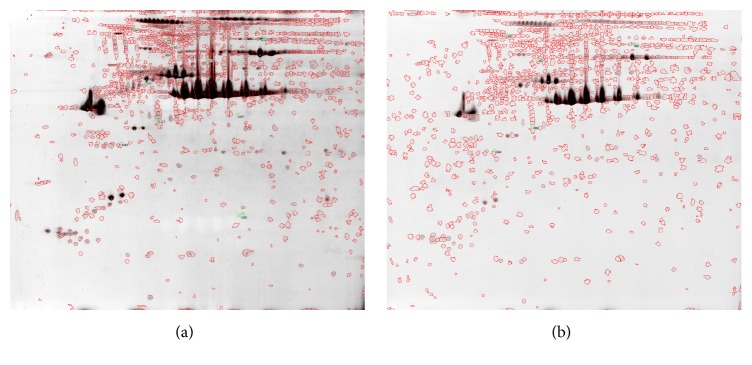
Comparison of two-dimensional (2D) gel electrophoresis results using colonic tissues between STC and control groups. STC group (a) and control group (b) 2D gel electrophoresis.

(1) Two-dimensional electrophoresis in proteomics research find that there were 14 differential protein spots between the two groups and the expression difference were more than 10 times (Figure 1).

(2) Mass spectrum (MS) identification and database search: after the protein spots with more than 10-fold difference between two groups were removed for the gels, they were subsequently dealt with using the following procedures including enzyme digestion, extraction of the peptides, and sample desalination. Finally, peptide mass fingerprint (PMF) and MS/MS spectrum were achieved through MALDI-TOF-MS analysis of proteins.

**Figure 2:**
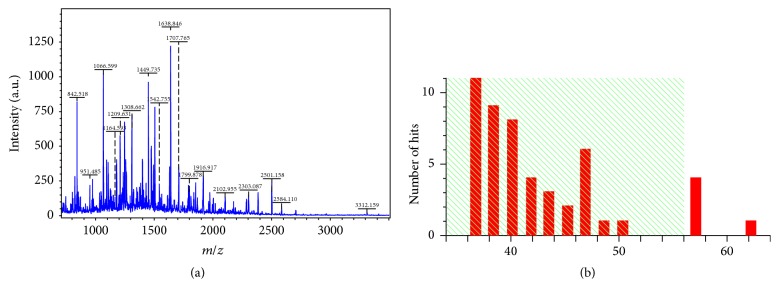
Mass spectrum identification figure of protein MRP4 and its matched analysis diagram. Mass spectrum identification figure of protein MRP4 (a) and its matched analysis diagram (b).

The retrieval for mass spectrometric data in Swiss-Prot successfully identified anterior 14 spots, wherein the expression of 12 spots is up and that of 2 spots is down in STC group (Tables 1 and 2). Our further studies show that multidrug resistance-associated protein 4 (MRP4) is of clinical significance (Figure 2). MRP4 is playing a critical role in the process of maintaining the stability of smooth muscle cells (SMC) [16, 17]. The downregulation of MRP4 expression in colon tissue of STC patients may be related to the decrease of SMC and may play a role in the pathogenesis of STC by affecting colon transmission.

## 4. Discussion

STC pathogenesis and mechanism not yet completely expounded that, at present clinical STC, patients mainly depend on purgative and gastrointestinal prokinetic agents for conservative management. A few clinical studies have confirmed that pharmacotherapy is effective in treating STC, but most patients with STC to accept drug treatment do not quite approve of the effect. The effectiveness of current STC drugs is fairly limited. The abuse of laxatives to assist defecation of patients with STC in clinical is very common; some patients who do not respond to conservative management are considered candidates for surgery [11].

Proteomics has been used to study the specific protein group that is functioning on different space and time and the study included three dimensions: expression proteomics, functional proteomics, and structural proteomics. Through the former three dimensions of study, researchers explore the function mechanism, regulatory mechanism, and interaction mechanism within group of corresponding proteins at the level of protein, which provide a theoretical reference for clinical diagnosis, pathologic study, drug screening, and pathogenesis of disease. STC currently faces many challenges, ranging from the elucidation of its pathophysiology to the effective treatment in patients. Proteomics has been widely employed in many diseases in the search of biomarkers, particularly cancer proteins. It has great potential to improve both our understanding and clinical management of STC. In the field of surgical research, proteomics studies mainly focus on tumor-related diseases at present; the proteomics studies for functional diseases are quite few and the proteomics study for STC is not reported so far. In our proteomics studies, we find that, compared with control group, the expression of 12 protein spots increased markedly and that of 2 protein spots decreased significantly in STC group. Among the low expression of proteins of STC, multidrug resistance-associated protein 4 (MRP4) is of clinical significance.

MRP4 belongs to transmembrane protein family; its main function is to transport intracellular cyclic nucleotides that include cyclic adenosine monophosphate and cyclic guanosine monophosphate. Some studies have confirmed that MRP4 participated in cellular excretion of resveratrol 3-O-glucuronide and resveratrol 4′-O-glucuronide [18], the inhibition of urinary excretion of methotrexate [19], cellular excretion of the raloxifene sulfates in breast cancer patients [20], the process of human obstructive cholestasis [21], and so on. Recently studies have revealed that MRP4 can be involved in maintaining the homeostasis of smooth muscle cell (SMC) by the regulation of intracellular cyclic nucleotide levels on the intracellular cyclic nucleotide signaling pathways (PKC, PKA, etc.). When the level of intracellular MRP4 is decreasing or inhibited, the significant reduction of the stability of SMC results in the decrease of proliferation cells and increase of apoptosis, which may be involved in the pathogenesis of idiopathic pulmonary arterial hypertension (IPAH) and coronary heart disease (CHD) [16, 17]. Our previous studies about STC have shown that, compared with normal colonic tissue, the number of SMC decreases and the apoptosis of SMC increases in colonic tissue with STC, which may be involved in the pathogenesis of STC. In this study, the homeostasis of SMC in colonic specimens of STC patients is changed and the decrease of proliferation cells and increase of apoptosis may result from the reduction of MRP4 expression, which may play a role in the pathogenesis of STC.

The results suggest that the different expression of proteins between STC and normal group may be associated with the pathogenesis of STC. The pathogenesis mechanism of STC remains largely obscure, which may result from multifactor; the change of the expression of MRP4 may play a certain role. Further studies for the relationship between MRP4 and the pathogenesis mechanism of STC and targeted drugs to the change of MRP4 are required.

## Figures and Tables

**Table 1:** Elevated expression proteins in colonic tissues of STC patients compared with control group.

Name	Score	pI	Formula weight	Fold change
ACTG	82	5.31	42108	17.91
ENPL	80	4.76	92696	29.30
F102B	80	6.62	39911	11.90
GBB1	76	5.60	38151	15.98
GELS	80	5.90	86043	13.72
K1C9	63	5.14	62255	20.01
MTMR7	62	5.94	76754	15.92
PDIA1	91	4.76	57480	22.71
PDIA6	68	4.95	48490	30.79
PFD6	78	8.83	14574	10.21
PRVA	60	4.98	12051	15.31
TRFE	79	6.81	79294	17.31

**Table 2:** Decreased expression proteins in colonic tissues of STC patients compared with control group.

Name	Score	pI	Formula weight	Fold change
MRP4	99	8.41	150344	−32.86
VIME	81	5.06	53676	−12.39
